# Guilty as Charged: The Role of Undercoordinated Indium in Electron-Charged Indium Phosphide Quantum Dots

**DOI:** 10.1021/acsnano.3c07029

**Published:** 2023-09-15

**Authors:** Maarten Stam, Indy du Fossé, Ivan Infante, Arjan J. Houtepen

**Affiliations:** † Optoelectronic Materials Section, Faculty of Applied Sciences, Delft University of Technology, Van der Maasweg 9, 2629 HZ Delft, The Netherlands; ‡ BC Materials, Basque Center for Materials, Applications, and Nanostructures, UPV/EHU Science Park, Leioa 48940, Spain; ¶ Ikerbasque, Basque Foundation for Science, Bilbao 48009, Spain

**Keywords:** quantum dots, DFT, electronic charging, undercoordination, trap states

## Abstract

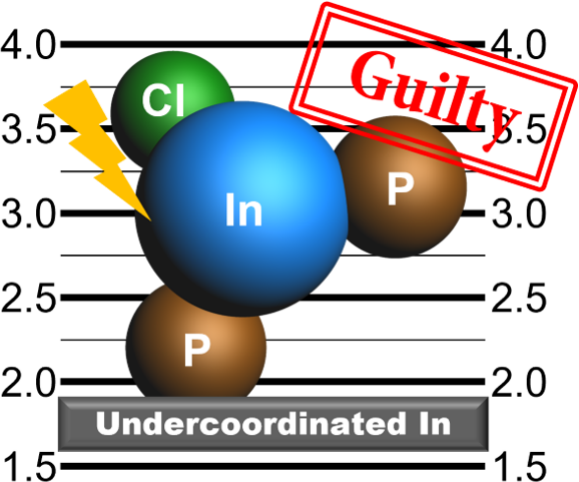

Quantum dots (QDs) are known for their size-dependent optical properties, narrow emission bands, and high photoluminescence quantum yield (PLQY), which make them interesting candidates for optoelectronic applications. In particular, InP QDs are receiving a lot of attention since they are less toxic than other QD materials and are hence suitable for consumer applications. Most of these applications, such as LEDs, photovoltaics, and lasing, involve charging QDs with electrons and/or holes. However, charging of QDs is not easy nor innocent, and the effect of charging on the composition and properties of InP QDs is not yet well understood. This work provides theoretical insight into electron charging of the InP core and InP/ZnSe QDs. Density functional theory calculations are used to show that charging of InP-based QDs with electrons leads to the formation of trap states if the QD contains In atoms that are undercoordinated and thus have less than four bonds to neighboring atoms. InP core-only QDs have such atoms at the surface, which are responsible for the formation of trap states upon charging with electrons. We show that InP/ZnSe core–shell models with all In atoms fully coordinated can be charged with electrons without the formation of trap states. These results show that undercoordinated In atoms should be avoided at all times for QDs to be stably charged with electrons.

## Introduction

Quantum dots (QDs) have size-dependent optical properties, narrow emission bands, and high photoluminescence quantum yields (PLQYs). These properties make QDs interesting candidates for optoelectronic applications, including photovoltaics, light-emitting diodes, and lasers.^1−10^ In particular, InP QDs are of commercial interest since the material is considered less toxic than Cd chalcogenide and Pb halide perovskite QD materials, making it more suitable for consumer applications.

InP-based QDs with high PLQY and narrow full width at half-maximum (fwhm) are nowadays synthesized and used in electronic devices.^6,11−14^ A common element in these devices is that charging of the materials with electrons and holes is required, through either electrical charge injection, intentional electronic doping, or photoexcitation.^2,15−27^ The simplest picture is that this results in the addition of charges to the conduction band (CB) or valence band (VB) states. Possibly present trap states in the bandgap would also simply be filled or emptied upon doping. However, it is well known that charging of semiconductors can result in the formation of charge-compensating defects.^28^ For semiconductor nanocrystals such defects most likely appear on the surface in the form of local reduction/oxidation of surface atoms or the formation of dimers.^29^ For example, Du Fossé *etal*. showed in a computational study that Cd-based QDs without dangling bonds are stable up to a charge of one electron but form trap states after injection of more electrons.^29,30^ Additionally it was shown that the local geometry of Cd atoms determines whether the reduction of a Cd atom is energetically favorable or not: absence of ligands results in reduction, and the presence of L-type ligands prevents reduction.^29,30^ Localized energy states in the bandgap are also observed for PbS QDs after the injection of three or four electrons, originating from undercoordinated Pb atoms leading to dimers on the surface.^31,32^ However, to the best of our knowledge, there is no atomistic understanding of the effects of charging InP QDs.

This work provides theoretical insight into electron charging of InP core-only and InP/ZnSe core/shell QDs. The influence of additional electrons on the structure and the electronic states of the QDs is studied. In line with the current understanding of the surface of InP QDs the QD models used in this study are all cation rich and contain negatively charged surface ligands, which compensate the positive charge from the excess cations on the surface.^33−38^ The structure and density of states (DOS) are first determined by density functional theory (DFT) calculations for the neutral QD, and consecutively the QD is subjected to electron charging. The method for simulating electron charging is adapted from the work of Du Fossé *etal*. and consists of the placement of one or more neutral K atoms on the surface of the QD.^29^ Per K atom, one electron is donated to the QD, resulting in a negatively charged QD and a positively charged K atom, while the overall system is charge neutral. DFT calculations are then performed to determine the effect of the additional electron in the QD.

Initially, DFT calculations, simulating electrochemical electron charging, are performed on InP core-only QDs with either a spherical or tetrahedral shape. The introduction of an electron into the QDs results in the formation of a trap state for both shapes. This trap is associated with a single undercoordinated surface In atom, which gets reduced upon electron addition, for both spherical and tetrahedral InP QDs.

A well-known solution to passivate the surface of QDs and thus creating fully coordinated atoms at the surface is the growth of an epitaxial shell on the QD core.^20,39^ To study the role of undercoordinated In atoms, two types of core/shell InP/ZnSe QDs are simulated in this study. One type contains undercoordinated In atoms, and in the other type, all In atoms are in a 4-fold coordination. Only the core/shell QDs with all In atoms in a 4-fold coordination have a trap state free bandgap after electron charging, even up to the addition of six electrons. Thus, the presence of undercoordinated In invariably leads to trap states when additional electrons are introduced in the QD. This shows that undercoordinated In atoms are not innocent but guilty as charged and responsible for charge compensation and trap state formation upon electron addition.

## Results and Discussion

### InP Core-Only Spherical QDs

**Figure 1:**
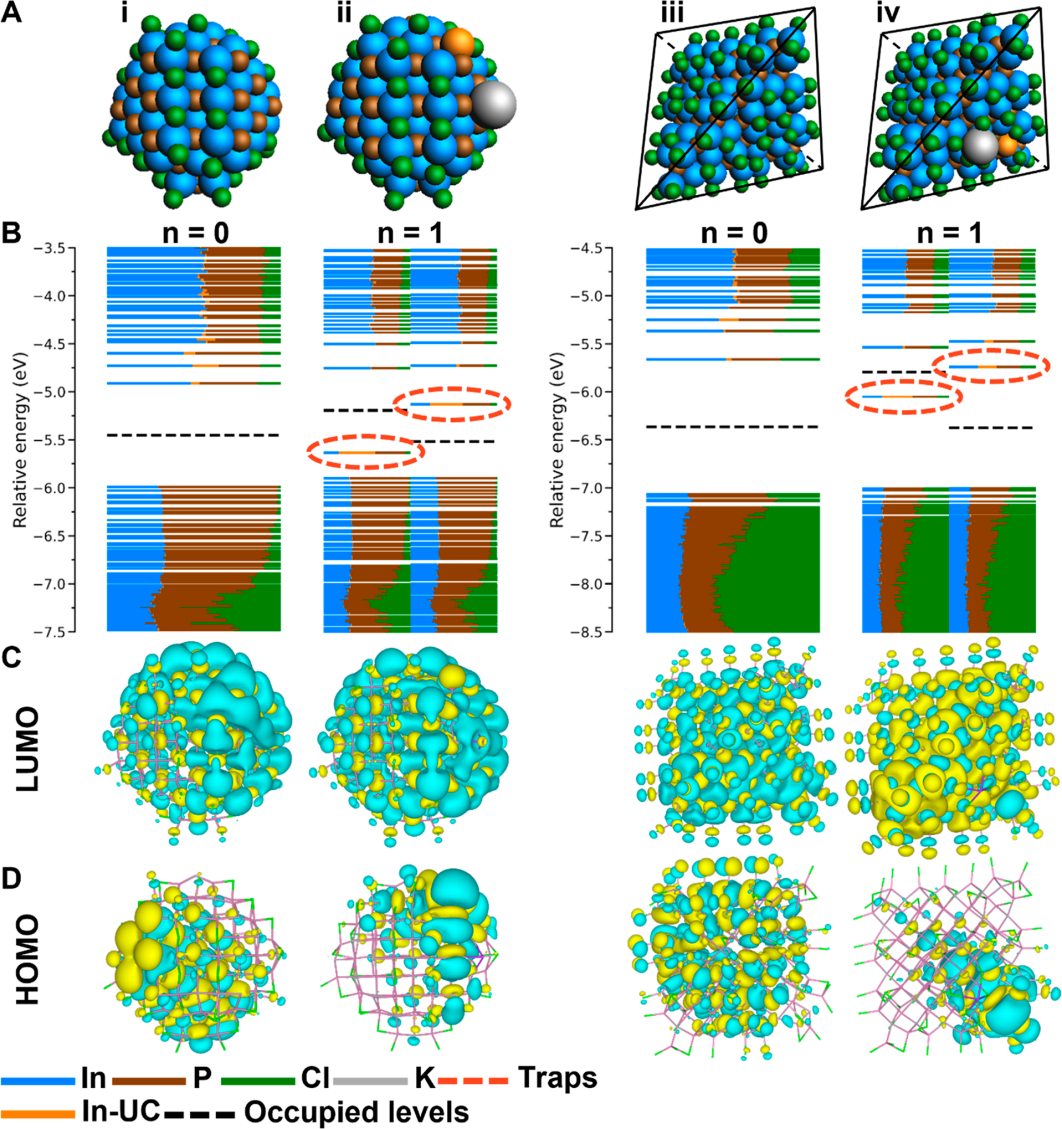
Charging of spherical and tetrahedral InP QDs. (A) Structures of the QD models where *n* indicates the number of injected electrons in the system. The black lines indicate a perfect tetrahedral shape. (B) The DOS for each of the QD models. Each horizontal line depicts an MO where the color indicates the fractional contribution to the MO of the corresponding element. The dashed black line indicates to which energy the MOs are filled with electrons. In the case of an odd number of electrons in the system (*n* = 1), the unrestricted calculation results in separated spin-up (α) and spin-down (β) orbitals, which are shown separately in the graph. Trap states are indicated by a dashed red circle. (C) Contour plots of the LUMO of the QDs or the LUMO of the α MOs for QDs with *n* = 1. (D) Contour plots of the HOMO of the QDs or the HOMO of the α MOs for QDs with *n* = 1.

InP QDs are reported with various shapes, but spherical and tetrahedral QDs are most commonly observed. The work of Dümbgen *etal*. predicts that both shapes are possible for small InP QDs but that at larger sizes only tetrahedral-shaped InP QDs allow for complete surface passivation.^35^ The requirement of charge balance requires three negatively charged ligands per excess In atom, and steric hindrance prevents this in large spherical QDs. In this work, both relatively small spherical and tetrahedral InP QDs are modeled and charged with electrons to determine and compare their stability upon electron charging. This section describes the results of electron charging InP core-only spherical QDs. Figure 1A-i shows the uncharged spherical InP QD, which is a zincblende In_68_P_55_Cl_39_ nanocrystal with a diameter of 1.9 nm. Figure 1B-i shows the DOS for this QD. It exhibits a bandgap that is free of electronic states and with all VB levels filled with electrons, which are below the dashed black line, and all CB levels empty, which are above the dashed black line. Contour plots of the lowest unoccupied molecular orbital (LUMO) and the highest occupied molecular orbital (HOMO) are shown in Figure 1C-i and D-i, respectively. Both the LUMO and HOMO are delocalized molecular orbitals (MOs), indicating that the VB and CB edges are not localized trap states. This shows that this QD model results in a trap-free bandgap, in line with similar results on Cd-based QDs and recent results on InP QDs.^29,33,35^

After establishing that the spherical InP QD model corresponds to a trap-free system, we investigated what happens upon electron charging. Figure 1A-ii shows the InP QD with an additional K atom, which results in the injection of one electron in the QD. It is observed that one In atom is ejected from the lattice at the surface of the InP model, shown in orange, indicating a structural change in the model. A close-up of this In atom in both the neutral QD and the electron charged QD is shown in Figure S1. Figure 1B-ii shows the DOS for this QD separated into the DOS for the spin-up (α) MOs and spin-down (β) MOs, as a result of the unrestricted calculation used for an odd number of electrons.^29^ The DOS in Figure 1B-ii shows trap states in the bandgap at −5.63 and −5.12 eV for the α and β MOs, respectively. The contribution of the ejected In atom to this trap state is 42% and 38% for the α and β MOs, respectively, indicating that this In atom is mainly responsible for the formation of the trap state. The energy distribution of the DOS for the spherical InP QD for *n* = 0 and *n* = 1 is shown in Figure S2. The contour plots of the MO of the trap state, the HOMO of the α MOs, and the LUMO of the β MOs are shown in Figure 1C-ii and in Figure S3 in the Supporting Information, respectively. These contour plots indicate the localization of the trap state MO on the ejected In atom. The LUMO of the α MOs remains delocalized, as shown in Figure 1D-ii, showing that the CB edge has not changed. From these results, it is concluded that the ejected atom is responsible for the formation of the trap state.

To understand why this particular In atom forms a trap state after electron addition, the coordination number of the In atoms in the QD is calculated. The geometry optimized model of the InP spherical QD has In–P and In–Cl bond lengths of 2.53 to 2.65 Å and 2.34 to 2.73 Å, respectively. The coordination number for every atom is therefore calculated by determining the number of atoms within a radius of 2.75 Å. Following this definition, it was found that the QD has 16 In atoms with a 3-fold coordination, whereas the preferred coordination number for InP in the zinc-blende lattice is four. These undercoordinated atoms are all located on the surface of the QD, as can be seen in Figure S4 with the undercoordinated atoms in red. In accordance with literature, no trap states are formed by these 3-fold-coordinated atoms before the addition of extra electrons, as has already been shown in the DOS in Figure 1B-i.^33,35^ However, the atom that is ejected from the QD lattice after electron addition, as described above, is one of the undercoordinated In atoms. The fact that the trap state is localized on the undercoordinated In atom leads to the hypothesis that undercoordinated In atoms are responsible for trap formation when additional electrons are provided. Further charging of the QD with *n* = 2 does not lead to the creation of more trap states but to filling of the energy level of the trap state with two electrons, indicating that the same surface In atom gets further reduced. However, charging the QD with *n* = 3 results in an additional trap state in the bandgap, as displayed in Figure S5 in the Supporting Information. The additional trap state for the QD with *n* = 3 is localized on another undercoordinated In atom. Charging with *n* = 4 again does not lead to the formation of an additional trap state but rather to the filling of the second trap state with a second electron.

### InP Core-Only Tetrahedral QDs

To determine whether the formation of a trap state after the addition of an electron depends on the shape of the InP QD, tetrahedral QDs were also investigated. Figure 1A-iii shows a tetrahedral In_84_P_56_Cl_84_ QD with an edge diameter of 2.6 nm. The calculated DOS is shown in Figure 1B-iii and shows a bandgap that is free of trap states, and the contour plots shown in Figure 1C-iii and D-iii indicate delocalization of the LUMO and HOMO orbitals. Hence, similar to the spherical QD in Figure 1A-i, the tetrahedral QD model results in a trap-free bandgap.

To simulate the electron charging for the tetrahedral QD, one potassium atom is placed on the surface of the model, as shown in Figure 1A-iv. The resulting DOS is shown in Figure 1B-iv, and trap states within the bandgap are observed at energies of −6.04 and −5.73 eV for the α and β orbitals, respectively. A contour plot of the α trap level is shown in Figure 1D-iv and shows a localization of the wave function in one of the corners of the QD. The largest contribution, 35%, to this trap state comes from one In atom, and it is therefore concluded that this In atom is responsible for the formation of the trap state. The coordination number of this particular In atom is three, again indicating that undercoordinated In atoms are responsible for trap state formation upon electron charging.

Thus, for both the spherical- and tetrahedral-shaped InP QD models, it is an undercoordinated In atom that has a large contribution to the formation of a trap state after the injection of one electron. Interestingly, reduction of Cd in CdSe QDs only occurs after the addition of two electrons.^30^ The more facile reduction of In can be explained by its higher Pauling electronegativity of 1.78 compared to cadmium with an electronegativity of 1.69 and is in line with the more positive standard reduction potential for In^3+^ reduction (In^3+^ + 3e^–^ → In; *E*^0^ = −0.34 V vs NHE) than for Cd^2+^ reduction (Cd^2+^ + 2e^–^ → Cd; *E*^0^ = −0.40 V vs NHE),^40−42^ although it should be noted that these reduction potentials depend on the solvation energy of the ions in water and represent the reduction of free metal ions not the semiconductor. The formation of a trap state after injection of one electron suggests that in the presence of excess electrons trap states form due to undercoordinated atoms independent of the shape of the InP QD, suggesting that it is mostly the local coordination that determines the stability of In atoms against reduction. The role of undercoordinated In also suggests that trap state formation can be prevented by ensuring full coordination of every atom.

Such full coordination can be achieved by growing an epitaxial shell around the core with a different material that has the same crystal structure and a matching lattice constant. Currently, the best InP-based QDs that are reported in literature are InP/ZnSe/ZnS core/shell/shell particles with PLQYs reaching unity.^6,12,14,43,44^ The result of shelling with ZnSe or ZnS is that the QDs are terminated with Zn atoms instead of In atoms. The Pauling electronegativity of Zn is 1.65, which is lower than both In (1.78) and Cd (1.69). In line with this, the standard reduction potential of Zn^2+^ is much more negative (*E*^0^ = −0.76 V vs NHE), so it is expected that Zn ions are more stable under reductive conditions. In both experimental and computational studies it is indeed found that Zn-terminated particles have higher electrochemical stability for both CdSe/CdS/ZnS and InP/ZnSe/ZnS QDs.^20,23,29^ With both the complete coverage of the core In atoms and the increased stability in mind, InP/ZnSe core/shell QD models are developed for this study.

### InP/ZnSe Core/Shell QDs

The effect of passivating surface In with the ZnSe shell is tested by first constructing a QD with an incomplete monolayer, leaving some surface In exposed and undercoordinated. Such QDs with thin/incomplete shells have recently also been described in experimental literature.^45^ Next we added an additional complete ZnSe monolayer to fully coordinate all In atoms and to simulate QDs with a thicker shell.

The exact composition of the incomplete shell is not chosen randomly, but corresponds to the introduction of specific surface vacancies which are known to occur on bulk III–V and II–VI semiconductor surfaces and prevent the formation of surface bands.^46^ We are currently preparing a manuscript on this topic. Here the surface reconstruction that we introduce is the removal of 25% of the surface Zn atoms from the (111) facets. Figure S6 shows a schematic example of the surface reconstruction. Charge balance of the model is achieved by adapting the number of surface Cl atoms. Such cation vacancies have earlier been shown to lead to delocalized HOMO and LUMO levels for CdSe QDs by Vozzny and Sargent.^47^

**Figure 2:**
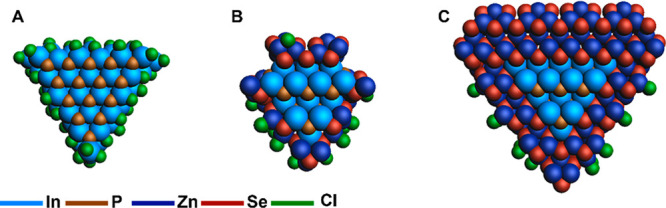
Two-dimensional cross-sections of the tetrahedral core and core/shell QD models. (A) InP core, (B) InP/ZnSe(1 ML) and (C) InP/ZnSe(2 ML).

The two InP/ZnSe core/shell QDs developed for this study both include surface reconstructions and are based on tetrahedral shapes. The tetrahedral-shaped QDs are chosen because the work of Dümbgen *etal*. shows that larger InP QDs prefer a tetrahedral shape to produce sufficient surface area for all required ligands.^35^ Cross-sections of the InP core-only and InP/ZnSe core–shell QDs are shown in Figure 2. Figure 2B shows an In_31_P_20_Zn_72_Se_72_Cl_33_ QD which has one monolayer of the ZnSe shell, including surface reconstructions, and is called InP/ZnSe(1 ML) for simplicity. Note that for this QD the four corner In atoms of the core are removed, which is because they were found to be unstable during the geometry optimization calculations. The surface reconstructions lead to an incomplete ZnSe shell, allowing to test our hypothesis that the presence of undercoordinated In atoms will lead to the formation upon charging of the QD with electrons. Figure 2C shows the In_35_P_20_Zn_322_Se_328_Cl_33_ QD with two monolayers of ZnSe shell named InP/ZnSe(2 ML) and has the surface reconstructions on the outermost ZnSe layer. Due to the second layer of ZnSe, all In atoms have a 4-fold coordination, which should lead to a trap-free system upon charging with electrons, according to our hypothesis. The results of the addition of electrons to these QDs are discussed in the following sections.

**Figure 3:**
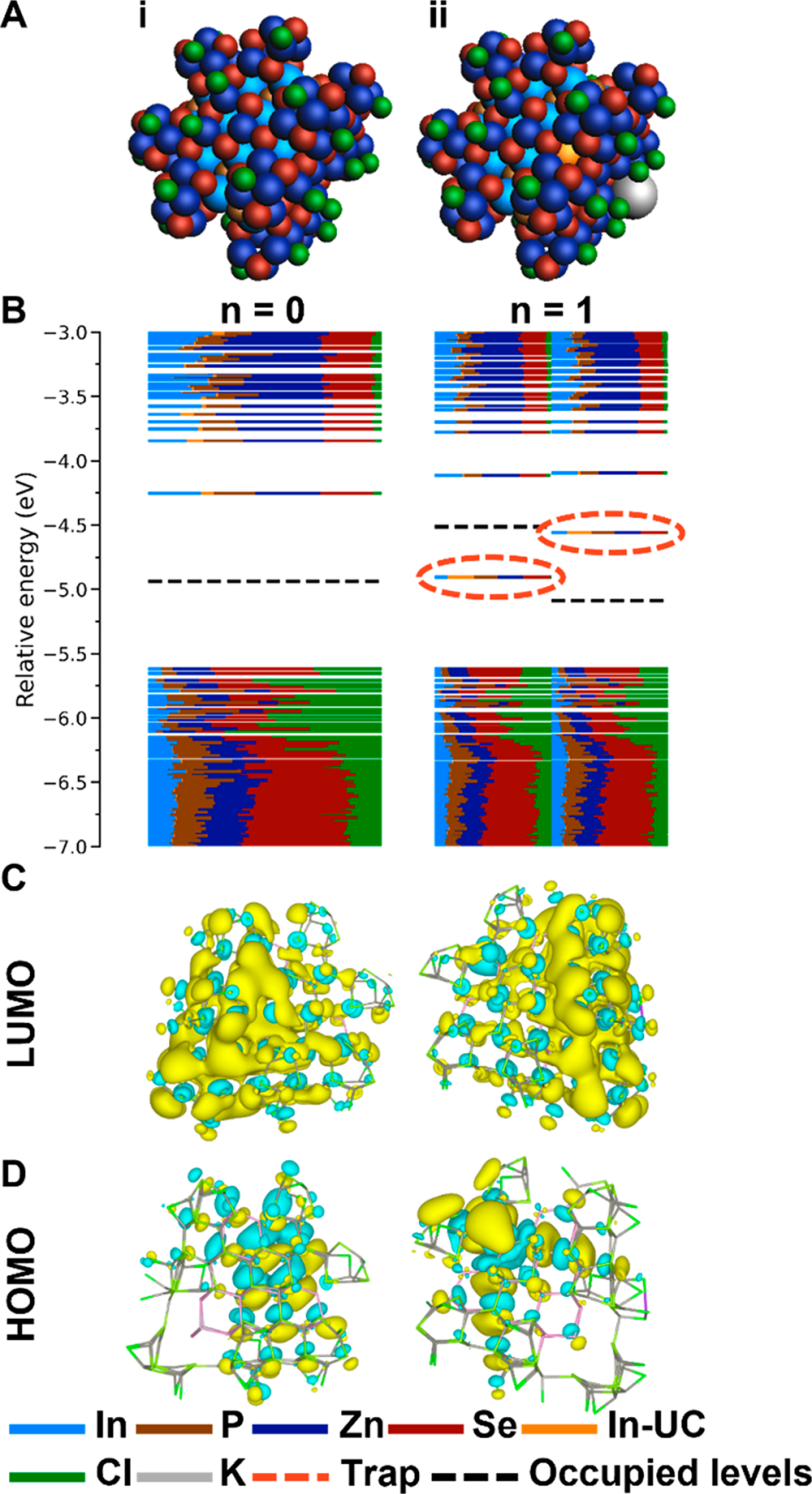
Charging of the InP/ZnSe(1 ML) QD. (A) Structures of the QDs where *n* indicates the number of injected electrons in the system. (B) The DOS for both QDs. The trap states are indicated with a dashed red circle. (C) Contour plot of the LUMO of the QD for *n* = 0 and the LUMO of the α MOs for *n* = 1. (D) Contour plot of the HOMO of the QD for *n* = 0 and the HOMO of the α MOs for *n* = 1.

The InP/ZnSe(1 ML) QD is shown in Figure 3A-i, and the cross-section of this model is shown in Figure 2B. The model is created by taking an InP core QD and adding one epitaxial layer of ZnSe. Subsequently, surface reconstructions are performed on the ZnSe layer, meaning that 25% of the surface Zn atoms are removed to create vacancies in the pattern as shown in Figure S6. However, the result of the surface reconstruction is that not all In atoms have a 4-fold coordination.

The DOS for the InP/ZnSe(1 ML) QD is shown in Figure 3B-i. It features a bandgap of 1.36 eV, clear of localized states, and evident contribution of Zn and Se to all MOs is observed. The LUMO, displayed in Figure 3C-i, is delocalized over both the core and the shell atoms. In Figure 3D-i, the HOMO of the core/shell QD is shown, and the MO is mostly delocalized over the shell atoms, which is in agreement with the relatively large contribution of Zn and Se observed in the DOS. A true type-I offset is not observed. This can be the result of the small size of the InP core but is likely also the result of electric fields arising at the core–shell interface.^48^ Therefore, it is difficult to draw conclusions about the energy offset between the core and the shell. The coordination number for all In atoms is determined for this model, and it is found that four In atoms are undercoordinated. Similar to the InP core, these undercoordinated In atoms do not result in trap states for the QD without excess electrons.

The addition of electrons is again simulated by placing a potassium atom on the surface of the InP/ZnSe(1 ML) QD. Figure 3A-ii shows the structure of the InP/ZnSe(1 ML) QD core/shell QD charged with a single electron (and a single potassium cation for charge compensation). The corresponding DOS, shown in Figure 3B-ii, contains in-gap trap states at −4.91 and −4.56 eV for the α and β orbitals, respectively. A contour plot of the in-gap trap state of the α orbitals is shown in Figure 3D-ii. The MO is significantly localized on one of the In atoms of the InP core and is colored orange in Figure 3A-ii. The contribution of this particular In atom to the MO is 23%, shown in orange in the DOS, indicating that the state is significantly localized on this In atom. The In atom responsible for the trap state has a 3-fold coordination. These results show that the presence of an incomplete ZnSe shell is not sufficient to prevent trap state formation after electron addition if undercoordinated In atoms are present.

**Figure 4:**
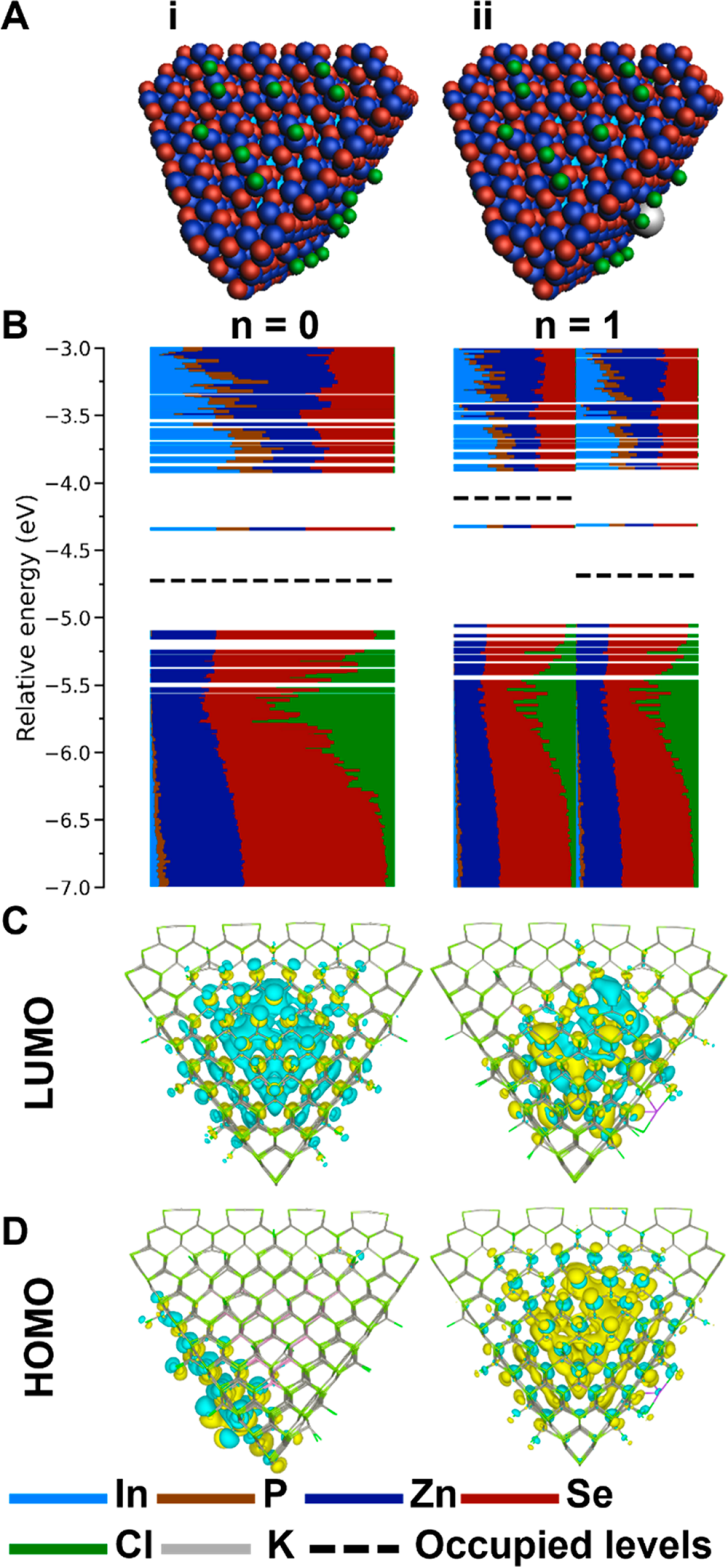
Charging the InP/ZnSe(2 ML) QD. (A) Structures of the QDs where *n* indicates the number of injected electrons in the system. (B) The DOS for both QDs. (C) Contour plot of the LUMO of the QD for *n* = 0 and the LUMO of the α MOs for *n* = 1. (D) Contour plot of the HOMO of the QD for *n* = 0 and the HOMO of the α MOs for *n* = 1.

To prevent undercoordinated In atoms, a second layer of ZnSe is added to the QD model, resulting in InP/ZnSe(2 ML). The cross-section in Figure 2C and the full QD in Figure 4A-i display that all In atoms of the InP core are covered by the ZnSe shell layers. The DOS calculated for this QD is shown in Figure 4B-i, and a bandgap of 0.77 eV is observed. Although the LUMO has a relatively large energy difference with the other MOs in the CB, the energy state is clearly delocalized over the entire QD, as shown in Figure 4C-i. The HOMO is delocalized over several shell atoms, indicating that it is not a trap state level, as shown in Figure 4D-i. Moreover, all In atoms are 4-fold coordinated, meaning that there are no undercoordinated In atoms present.

With all In atoms 4-fold coordinated, the InP/ZnSe (2 ML) QD is subjected to the addition of an extra electron. The QD with the extra potassium atom on the surface is visible in Figure 4A-ii, and the corresponding DOS is plotted in Figure 4B-ii. The DOS shows a bandgap of 0.74 eV, which is clear from gap trap states. The HOMO of the α set of the orbitals is shown in Figure 4D-ii and displays delocalization of the energy state over the entire QD, indicating that the filled orbital is not a trap state but that the electron is injected into the CB. To study the stability of the InP/ZnSe(2 ML), the QD was charged with up to six electrons and no trap states were formed; see Figure S7 in the Supporting Information. These results confirm that a 4-fold coordination due to two layers of a ZnSe shell prevents formation of trap states when electrons are added to the QD. The work of Park *etal*. shows that electrochemical charging of InP-based QDs is based on QDs with complete coverage of ZnSe/ZnS shells.^23^ However, there is no discussion on In reduction on the surface of InP core-only QDs after electron charging. Passivation of the undercoordinated In atoms is here achieved by a complete ZnSe shell, but this might also be achieved by coordination to ligands. The exact effects of coordination of undercoordinated In atoms is outside the scope of this work, but the work of Du Fossé *etal*. shows that L-type ligands stabilize QDs against surface reduction, and it is likely that these results also hold for InP QDs.^30^

It is concluded from the results in this work that trap state formation after electron charging a QD can only be prevented by ensuring 4-fold passivation of all the In atoms in the QD. Implementation of InP-based QDs in electronic devices depending on electron charging of these QDs therefore requires attention to surface and interface coordination of the In atoms.

## Conclusion

In conclusion, this work describes the effect of adding electrons to InP core-only and InP/ZnSe core–shell QDs by DFT calculations. The results show that charging of InP QDs with electrons always results in the reduction of a surface or interface ion if undercoordinated In atoms are present. Only the formation of a complete ZnSe shell prevents this reduction. InP is thus intrinsically less stable against reduction than Cd-based QDs, but it can be stabilized with Zn chalcogenide shells, provided that all In atoms become fully coordinated.

## Methods

In agreement with previous work and the current understanding of the surface composition of InP QDs, the QD models in this work are cation-rich and have chloride anions on the surface to preserve charge balance.^33−38^ The chloride anions are electronically similar to the carboxylic acid ligands used in experiments but are computationally less expensive.^29,35,49^ To calculate the required number of chloride atoms and the number of excess electrons after charging, the charge-orbital model of Voznyy *etal*. is used, which is defined as
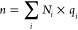
with *n* representing the number of excess electrons, *N*_*i*_ the number of atoms of type *i*, and *q*_*i*_ the most common oxidation state of atom type *i*.^50^ For *n* > 0, excess electrons are present in the QD and the QD is therefore negatively charged; for *n* < 0 the QD becomes positively charged. The oxidation states of the atom types used in the QD models of this work are assumed to be 3+, 2+, 1+, 3–, 2–, and 1– for In, Zn, K, P, Se, and Cl, respectively.

Calculations for structural relaxations, DOS, and MOs are all performed at the DFT level using the CP2K quantum chemistry software package.^51,52^ A Perdew–Burke–Ernzerhof (PBE) exchange–correlation functional and a double-ζ valence polarization basis set are used for all atoms.^53,54^ Effective core potentials from the GTH pseudopotentials account for scalar relativistic effects. Simulations were all performed at 0 K in the gas phase. For QD systems with an odd number of electrons, unrestricted spin calculations were performed. In unrestricted calculations, the spin-up (α) and spin-down (β) electrons are calculated independently from each other, resulting in separate densities of states and MOs. For all contour plots of MOs a value of 0.005 e/bohr^3^ was used.

It should be noted that the use of the PBE exchange–correlation leads to underestimation of the bandgap.^55^ The absolute energies of the MOs may therefore differ from the experimentally obtained values. The energy levels cannot directly be related to experiments, but the trends described in this work are expected to be valid.^29^

## Supporting Information Available

A close-up of the undercoordinated In atom in the spherical InP QD that is responsible for the trap state before and after charging; the DOS for the spherical core-only QD before and after charging; a table containing the energy of the conducting and valence band edge, the energy of the formed trap states, and the bandgap energy; contour plots for the β MOs of the spherical core only InP QD for *n* = 1; the structure of the spherical core-only QD with *n* = 0 with all undercoordinated In atoms indicated in red; the DOS for the spherical QD with *n* = 2, 3, and 4; a schematic drawing of the surface reconstructions; and the structure and the DOS of the InP/ZnSe(2 ML) QD with *n* = 6 (PDF)
