# Supplementary material for: Guilty as Charged: The Role of Undercoordinated Indium in Electron-Charged Indium Phosphide Quantum Dots
Source: ACS Nano. 2023 Sep 15;17(18):18576–83. doi: 10.1021/acsnano.3c07029 (PMC10540256; doi:10.1021/acsnano.3c07029)
Supplement: Supplementary file 1 — nn3c07029_si_001.pdf [file nn3c07029_si_001.pdf]

Supporting information for

Guilty as Charged: the Role of Undercoordinated Indium in Electron Charged Indium Phosphide Quantum Dots

Maarten Stam[†], *Indy du Fossé*[†], *Ivan Infante*[‡] *and Arjan J. Houtepen*[†]*

[†] Optoelectronic Materials Section, Faculty of Applied Sciences, Delft University of Technology, Van der Maasweg 9, 2629 HZ Delft, The Netherlands

[‡] Istituto Italiano di Tecnologia – IIT, 16163 Genova, Italy; BC Materials, Basque Center for Materials, Applications, and Nanostructures, UPV/EHU Science Park, Leioa 48940, Spain; Ikerbasque, Basque Foundation for Science, Bilbao 48009, Spain

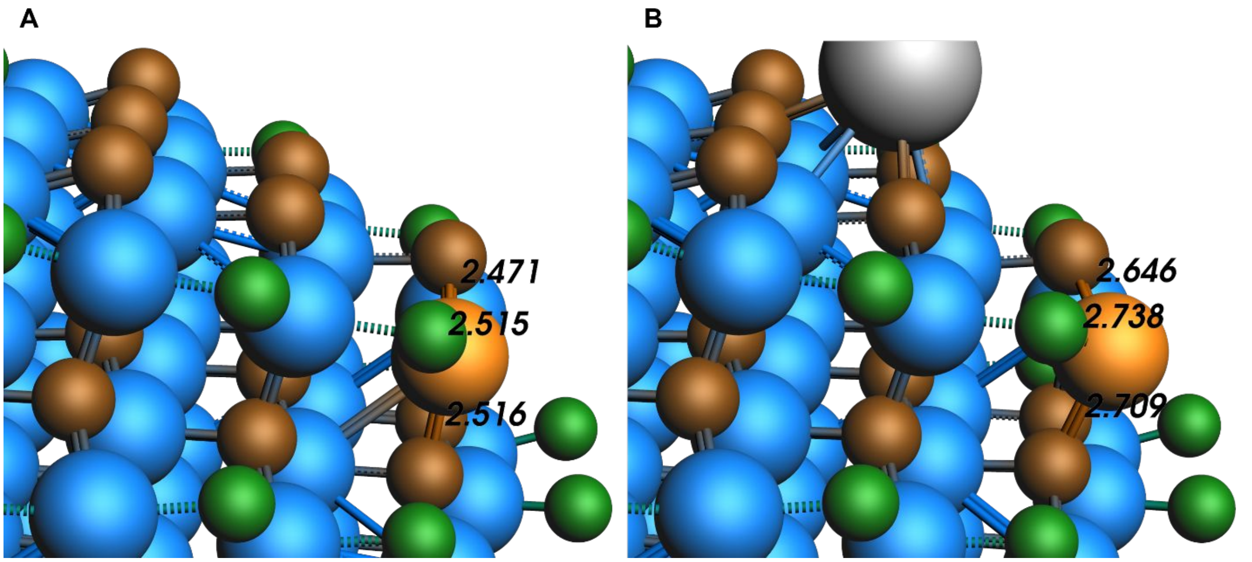

Figure S1. Close-up of the undercoordinated In atom in the spherical core-only InP QD that is responsible for the formation of the trap state after electron charging for the neutral QD in A) and for n = 1 in B). The undercoordinated In atom is colored in orange and the distance between the coordinating atoms in given in Å.

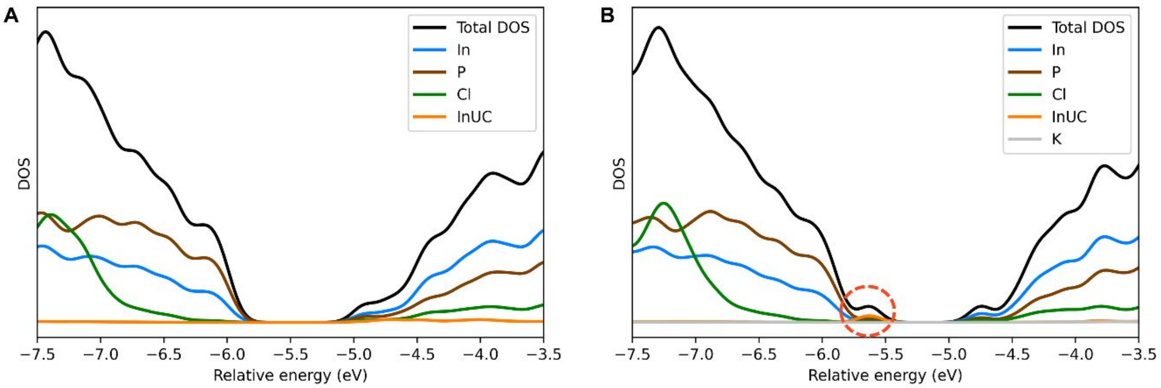

Figure S2. DOS for the spherical core-olny QD for n = 0 in A) and for n = 1 in B). The trap state is indicated by a red dashed circle.

Table S1. Relative energy levels for each used QD model of the valence and conduction band, the relative energy of the trap states and the bandgap energy.

	VB (eV)	Trap (eV)	CB (eV)	BG (eV)

Spherical InP n = 0	-5.999	N/A	-4.920	1.078
Spherical InP n = 1	-5.893	-5.627	-4.747	1.146
Tetrahedral InP n = 0	-7.061	N/A	-5.656	1.405
Tetrahedral InP n = 1	-7.003	-6.045	-5.530	1.473
InP/ZnSe(1ML) n = 0	-5.623	N/A	-4.258	1.365
InP/ZnSe(1ML) n = 1	-5.624	-4.911	-4.121	1.502
InP/ZnSe(2ML) n = 0	-5.115	N/A	-4.348	0.767
InP/ZnSe(2ML) n = 1	-5.060	N/A	-4.325	0.735

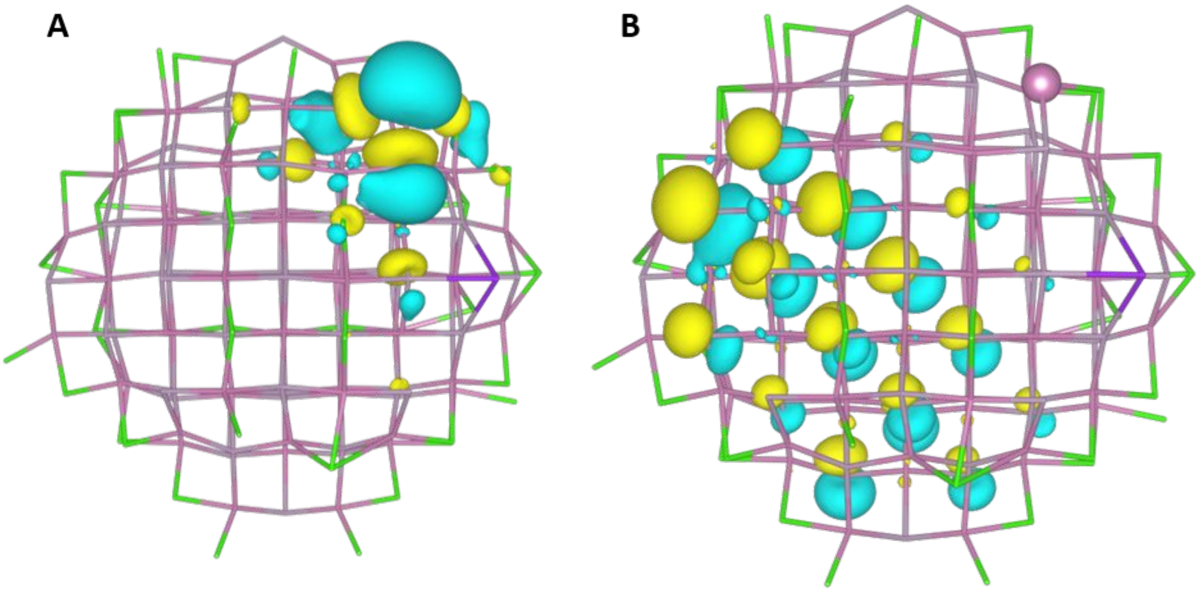

Figure S3. A) Contour plot of the LUMO of the β MOs of the spherical core-only InP QD for n = 1 using a contour plot value of 0.005 e/bohr^3. B) Contour plot of the HOMO of the β MOs of the spherical core-only InP QD.

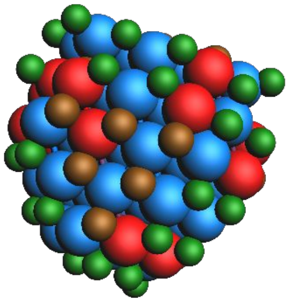

Figure S4. Structure of the spherical core-only QD with the undercoordinated In atoms in red.

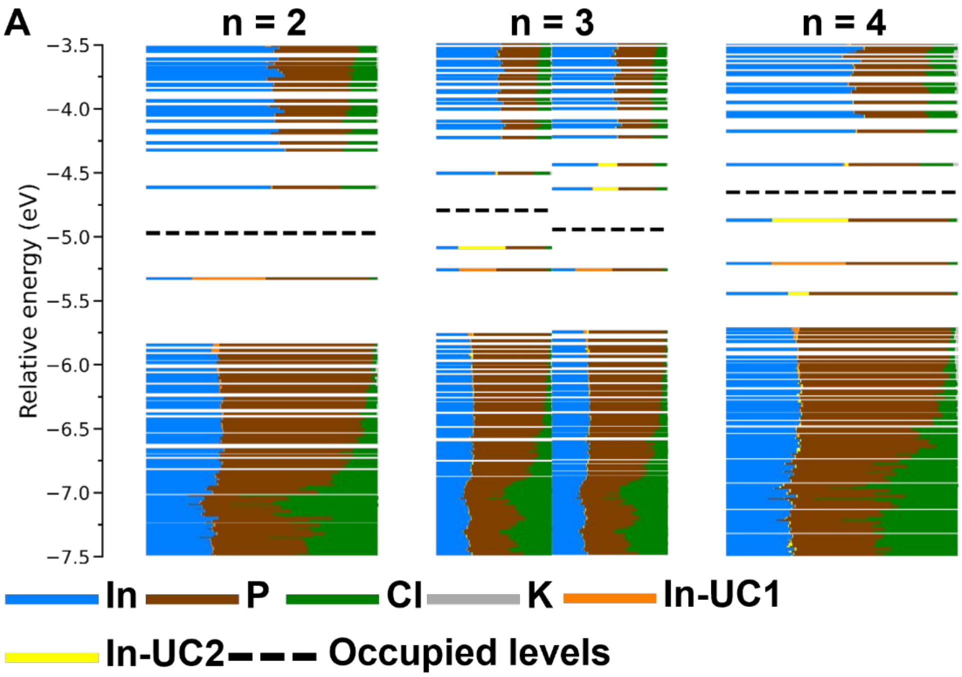

Figure S5. Charging of InP QDs with n = 2, 3 and 4. The second In atom that is reduced due to charging is indicated with a yellow color.

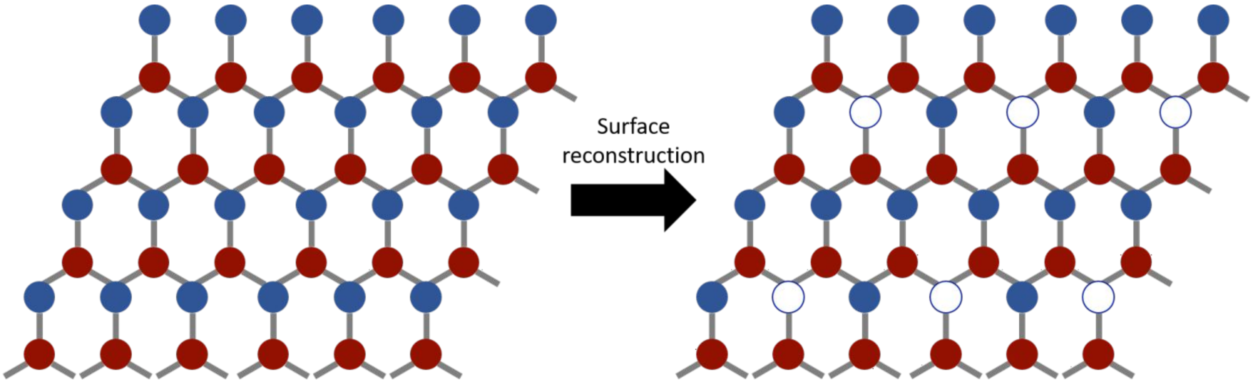

Figure S6. Schematic drawing of the surface reconstruction where cation vacancies are systematically introduced. The cations are depicted as blue spheres, the anions as red spheres and the introduced vacancies as white spheres with a blue outline.

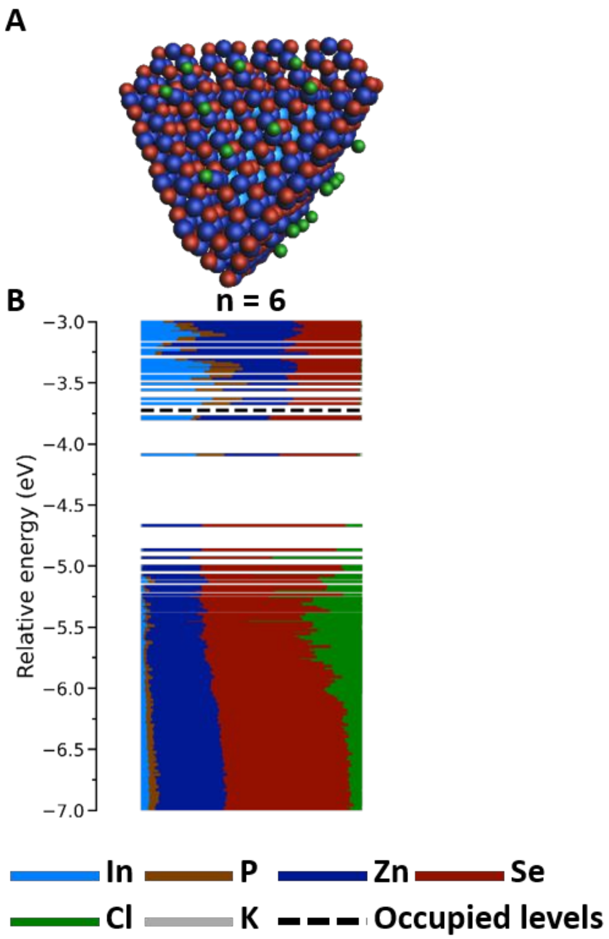

Figure S7. Charging of the InP/ZnSe(2ML) QD with n = 6. A) The structure of the QD with six potassium atoms on the surface. B) The DOS for the InP/ZnSe(2ML) with n = 6.
